# N6-methyladenosine demethylase FTO regulates synaptic and cognitive impairment by destabilizing PTEN mRNA in hypoxic-ischemic neonatal rats

**DOI:** 10.1038/s41419-023-06343-5

**Published:** 2023-12-13

**Authors:** Jianhui Deng, Yanling Liao, Jianghu Chen, Andi Chen, Shuyan Wu, Yongxin Huang, Haitao Qian, Fei Gao, Guixi Wu, Yisheng Chen, Xiaohui Chen, Xiaochun Zheng

**Affiliations:** 1 Department of Anesthesiology, Shengli Clinical Medical College of Fujian Medical University Fujian Provincial Hospital, Fuzhou, China; 2 Center for Experimental Research in Clinical Medicine, Fujian Provincial Hospital, 350001 Fuzhou, China; 3 Fujian Emergency Medical Center, Fujian Provincial Key Laboratory of Critical Care Medicine, Fujian Provincial Co-Constructed Laboratory of Belt and Road, Fuzhou, China

**Keywords:** Cognitive control, Transcriptomics

## Abstract

Hypoxic-ischemic brain damage (HIBD) can result in significant global rates of neonatal death or permanent neurological disability. N6-methyladenosine (m6A) modification of RNA influences fundamental aspects of RNA metabolism, and m6A dysregulation is implicated in various neurological diseases. However, the biological roles and clinical significance of m6A in HIBD remain unclear. We currently evaluated the effect of HIBD on cerebral m6A methylation in RNAs in neonatal rats. The m6A dot blot assay showed a global augmentation in RNA m6A methylation post-HI. Herein, we also report on demethylase FTO, which is markedly downregulated in the hippocampus and is the main factor involved with aberrant m6A modification following HI. By conducting a comprehensive analysis of RNA-seq data and m6A microarray results, we found that transcripts with m6A modifications were more highly expressed overall than transcripts without m6A modifications. The overexpression of FTO resulted in the promotion of Akt/mTOR pathway hyperactivation, while simultaneously inhibiting autophagic function. This is carried out by the demethylation activity of FTO, which selectively demethylates transcripts of phosphatase and tensin homolog (PTEN), thus promoting its degradation and reduced protein expression after HI. Moreover, the synaptic and neurocognitive disorders induced by HI were effectively reversed through the overexpression of FTO in the hippocampus. Cumulatively, these findings demonstrate the functional importance of FTO-dependent hippocampal m6A methylome in cognitive function and provides novel mechanistic insights into the therapeutic potentials of FTO in neonatal HIBD.

## Introduction

Hypoxic-ischemic brain damage (HIBD) is a major reason for neonatal brain damage and death [1]. The overall incidence of HIBD is ~0.1% [2, 3], though rates are higher (>10%) in some developing countries [4]. Major hippocampal system functions (i.e., cognition and memory) depend on synaptic transmission properties, and cognitive impairment is significantly correlated with hippocampal synapse loss [5, 6]. Depending on severity, HI may lead to delayed neurite outgrowth and synaptogenesis in animals during early development, resulting in long-term memory impairment [5]. Currently, there is a scarcity of clinical neuroprotective therapeutics, which underscores the immediate necessity to explore the underlying mechanisms of HIBD.

Autophagy is an intracellular degradation event that eliminates protein aggregates and abnormal organelles [7]. Normal functioning and homeostasis of the mammalian nervous system rely on proper autophagy [8, 9]. Neurodegenerative and neuropsychiatric disorders may be caused by dysfunctional autophagy [10–12]. Accumulating evidence indicates that autophagy activation is crucial in the pathogenesis of brain damage following neonatal HI [13, 14]. Further, aggravated autophagy accumulation eventually results in synaptic and cognitive impairment [15]. Inhibition of autophagy can considerably attenuate synaptic impairment and improve neurologic function in ischemic brain damage [16, 17]. As a positive regulator of this process, suppression of phosphatase and tensin homolog (PTEN) eliminates autophagy via the AKT/mTOR pathway [18, 19]. Accordingly, focusing on autophagy activation following neonatal HI may lead to development of promising therapeutic targets for improving synaptic impairment. Therefore, directing attention towards the activation of autophagy after neonatal HI injury may offer potential therapeutic targets for enhancing synaptic function.

The body of evidence regarding the role of epigenetics in various central nervous system biological processes and diseases continues to grow. Compared with other tissues, brain tissues show highly specific m6A methylome, suggesting that m6A is involved with regulation of brain-specific functions [20–22]. As it is the most abundant post-transcriptional modification in mRNA, m6A modification participates in many neuropathological and physiological processes, including neural development, Alzheimer’s disease, and stroke [23–25]. Thus, as aberrant m6A modification is a likely HIBD pathogenesis mechanism, we investigated the epitranscriptomic regulations underlying brain damage post-HI, identifying a predominant, novel mechanism contributing to synaptic and cognitive impairment via dysregulated RNA modifications.

As a major m6A demethylase, FTO is widely expressed and highly enriched in neurons, and can influence nervous system functions [26, 27]. FTO-deficient mice exhibit growth retardation, microcephaly, severe psychomotor delay, functional brain deficits, and facial dysmorphism, reflecting the importance of FTO in nervous system development and differentiation [28, 29]. Local FTO alterations in the developing brain are crucial for dynamic gene expression changes associated with synaptic plasticity and memory formation [30]. FTO also plays a key role in regulating autophagy, by targeting gene encoding autophagy-related proteins in various diseases [31–33]. However, there is limited knowledge regarding the possible role of FTO in regulating cognitive impairment induced by HI injury, as well as whether this protective effect may be associated with the inhibition of autophagy activation. Herein, we explored FTO regulation in neonatal HI-induced brain damage, to elucidate the mechanisms involved in this pathological process.

## Materials and methods

### Animals and ethics statement

Nursing Sprague Dawley (SD) rats and their offspring were obtained from Fujian Medical University. Rats were kept at 20–22 °C under a 12 h light–dark cycle with free food and water access. Experiments were conducted in accordance with the Guide for the Care and Use of Laboratory Animals the guidelines of the animal review committee at Fujian Medical University (protocol license number: IACUC FJMU 2022-0441).

### The establishment of HIBD model

The HIBD model was established by subjecting neonatal Sprague-Dawley (SD) rats at postnatal day (PND) 7 to unilateral carotid artery ligation under 3% isoflurane anesthesia (RWD, Shenzhen, China), followed by a 2 h exposure to 8% oxygen and 92% nitrogen at 37 °C. The chamber was maintained at 50–70% humidity using an atomizer. Sham-operated group rats were anesthetized and the left common carotid artery exposed, without ligation or hypoxia. Cohorts of rats were euthanized at 24 h, 48 h, 72 h, and 7 days post-HI.

### Primary hippocampal neuron culture

As previously described, primary hippocampal neuronal cultures were obtained from PND 0 rats [34]. Hippocampal tissues were dissected and then digested with papain (Macklin Inc, Shanghai, China) at 37 °C for 25 min. After filtration through a 70 µm cell strainer, the suspension was then centrifuged for 2 min. Cells were then seeded on poly-D-lysine-coated (Sigma-Aldrich) cover slips at a density of 1 × 10^6^ cells/well. A neurobasal medium (Sigma-Aldrich) containing B27 (Thermo Fisher Scientific, Waltham, MA) and 2 mM L-Glutamine (Thermo Fisher Scientific) was used for the cell culture. The basal medium was changed every 3–4 days by replacing half of it. The cultures attained a purity of >90% for primary hippocampal neurons.

### Oxygen-glucose deprivation and re-oxygenation (OGD/ R)

OGD/R was induced in primary hippocampal neurons as described below. Briefly, primary hippocampal neurons were initially maintained in an airtight chamber, equilibrated for 15 min with a continuous flux of gas (95% N2/5% CO2). The chamber was sealed and placed in an incubator for additional 3 h of OGD. primary hippocampal neurons were then re-oxygenated for applied time periods. Neuron cultured under normoxic conditions were used as a negative control [35].

### TTC staining

The presence or absence of infarction was evaluated by examining TTC-stained sections for the areas on the side of infarction that did not stain by TTC. After 48 h HI exposure, the brain was harvested and coronal brain slices were cut to 100 mm thickness. Incubation of 2,3,5-triphenyltetrazolium chloride (TTC, Sigma-Aldrich, Shanghai, China) for 15 min at 37 °C was performed for each section. ImageJ software was used to measure infarct area. Add the infarct volumes of all five sections to get the infarct volume of the entire brain. The calculation of infarct volume is determined using the following formula: [(volume of the normal hemisphere) - (volume of the non-infarct region in the affected hemisphere)] divided by the total hemisphere volume, multiplied by 100%.

### Nissl staining

Rats were anesthetized with isoflurane and euthanized by decapitation 48 h after HI injury. Sections were stained with 1% toluidine blue in distilled water. 5 mm sections were stained with Nissl dye (Beyotime Biotechnology, Shanghai, China), fixed with distilled water, preheated at 60 degrees Celsius for 20 min, and decolored with 95% ethanol. Neuronal loss was evaluated using Nissl-stained slices.

### m6A dot blotting analysis

Following the manufacturer’s instructions, total RNA was extracted with TRIzol (Invitrogen, Carlsbad, CA, USA). Then, an initial denature step at 100 °C for 1 min was followed by cooling on ice for 1 min. Equal amounts of total RNA were blotted on Hybond-N+ membranes (GE Healthcare, USA) and cross-linked with ultraviolet light. Blocking with PBST (5% nonfat milk and 0.1% Tween-20) was performed for 1 h, followed by overnight incubation with 1:1000 anti-M6A antibody (Synaptic Systems, USA) and secondary antibodies for 1 h at room temperature. Chemiluminescence was used to visualize bands. The membrane was stained with 0.02% methylene blue in 0.3 M sodium acetate at pH 5.2 to ensure equal loading.

### Immunofluorescence analysis

Deparaffinized paraffin sections were used for immunofluorescence staining. The sections were blocked for 30 min at room temperature with PBS containing 5% bovine serum albumin (BSA). For fluorescent double labeling with primary antibodies from different species, antibodies were applied simultaneously at 4 °C overnight. Then, sections were incubated with the respective secondary antibodies for 1 h at room temperature. Nuclei were counterstained with DAPI (Sigma Aldrich) for fluorescence microscopy. ImageJ software was used to calculate Pearson correlation coefficients.

In primary hippocampal neuron cultures, 4% paraformaldehyde was diluted in PBS and fixed for 20 min. Then, 0.3% Triton X-100 diluted in PBS was permeabilized for 5 min. After PBS washes, the fixed cells were blocked for 1 h at room temperature (3% BSA in PBS). A primary antibody incubation was performed overnight at 4 °C, followed by a fluorescent secondary antibody incubation at room temperature for 1 h. Nuclei were counterstained with DAPI (Sigma-Aldrich) for fluorescence microscopy. ImageJ software was used to calculate Pearson correlation coefficients.

### m6A RNA microarray

Using an Arraystar Super RNA Labeling Kit (Arraystar, MD, United States), cRNAs and immunoprecipitated RNAs were amplified and labeled separately with Cy5 and Cy3 fluorescent dyes. The RNeasy Mini Kit (Qiagen) was used to purify the RNA. After fragmenting the cRNAs at 60 °C for 30 min with 10X blocker and 25X fragmentation buffer, 55 L of 2X GE Hybridization buffer was added to dilute them. Mixtures of Cy3- and Cy5-labeled cRNA were hybridized to the Arraystar Epitranscriptomics Microarray. Hybridization was carried out at 65 °C for 17 h at a rotation speed of 10 rpm in an Agilent Hybridization oven (Agilent Technology, USA). Arrays were scanned using an Agilent G2505C Microarray Scanner (Agilent Technologies).

### RNA sequencing and bioinformatic analyses

The timepoint with the most obvious changes was determined by comparing m6A levels across six defined sham timepoints and five post-HI timepoints. Six rats each were selected from the HI (48 h) and sham (48 h) groups. The RNA from hippocampus tissues was purified using the TRIzol reagent (Invitrogen, CA, USA). Next, 1 ~ 2 ug of total RNA was employed for the depletion of ribosomal RNA (rRNA), following the Ribo-Zero™ rRNA Removal Kit (Illumina, San Diego, USA) guidelines. In accordance with the manufacturer’s instructions, a transcriptome library was generated using the KAPA-Stranded RNA-Seq Library Prep Kit (Illumina). Bioanalyzer 2100 (Aligent, Santa Clara, CA, USA) was used to assess RNA integrity. Solexa pipeline v1.8 (Off-Line Base Caller software) was used in the analysis and base calling of the images. Bioinformatics at Cambridge assessed the quality of all sequencing data using FastQC. Each sample’s transcript abundance was estimated with StringTie (1.3.4). FPKM was calculated at gene and transcript levels using R software Ballgown, and gene and transcript level expression was compared, after which differentially expressed genes were screened.

### Western blot analysis

According to the manufacturer’s instructions, RIPA Lysis Buffer (Beyotime Biotechnology) was used to extract proteins from rat hippocampal tissues. Electrophoretic separation by SDS-PAGE was carried out at 100 V for 60 min, followed by membrane transfer on PVDF at 100 V for 60 min. A primary antibody was incubated overnight at 4 °C, and a secondary antibody for 1 h at room temperature. Chemiluminescence was used to visualize bands (ECL Kit, Thermo Fisher Scientific). ImageJ software was used to analyze densitometry data to determine relative changes in protein expressions. Primary antibodies specific to the following proteins were used: Mettl14, FTO, YTHDF1, YTHDF3, PTEN, p-AKT, AKT, p-mTOR, mTOR, Beclin1, p62, LC3II, PSD-95, Synaptophysin, β-acitn (all from ABclonal, Wuhan, China), Mettl3, Wtap, and Alkbh5 (all from Abcam, Cambridge, UK).

### Real-time quantitative polymerase chain reaction

The total RNAs of primary hippocampal neurons were harvested using TRIZOL reagent (Invitrogen, Carlsbad, CA, USA). The RNA levels for PTEN were quantified by fast real-time PCR system (ABI 7500, Applied Biosystems, Carlsbad, CA, USA). Drawing dissolution curves based on experimental results determined gene amplification specificity. the 2-ΔΔ CT method was used to calculated the relative gene expression. PCR included the following sets of primers: F: CAAGATGATGTTTGAAACTAT, R: CCTTTAGCTGGCAGACCACAA. GAPDH was set as an internal control.

Actinomycin D (1 µg/mL; Sigma) was used to block transcription during chase-based mRNA half-life analysis, cells were harvested for RNA extraction and real-time quantitative polymerase chain reaction at t = 0, 3,6 h after actinomycin D treatment.

### Lentiviral transfection of primary cultured hippocampal neurons

A green fluorescent protein (GFP) label for a lentiviral vector plasmid system carrying the FTO gene was constructed by Shanghai Jikai Gene Technology Co., Ltd. The viral titer is >1E + 8 TU/ml. Primary cultured hippocampal neurons were transfected with lentiviral vectors at an appropriate multiplicity of infection according to the manufacturers’ instructions. The optimal MOI was detected according to the manufacturer’s instructions. Then, lentivirus vectors were transfected into the primary cultured hippocampal neurons with the optimal MOI, and the transfection efficiency was measured by the expression of the fluorescent protein. Thereafter, the expression levels of the FTO gene and protein were assessed via western blot.

### RNA interference

For in vitro RNA interference (RNAi), we purchased siRNA from HANbio (Shanghai, China) to specifically knock down the expression of the FTO gene in primary hippocampal neurons. Lipofectamine RNAiMAX (Invitrogen, Carlsbad, CA, USA) was used to transfect the cells with siRNA targeting FTO or scrambled siRNA as a control. After transfection for 48 h, FTO mRNA expression levels were determined using western blot. The sequences of si-FTO were as follows: sense, 5′GCAGCUGAAAUAUCCUAAATT3′, antisense, 5′ UUUAGGAUAUUUCAGCUGCTT 3′.

### MeRIP-qPCR

The m6A immunoprecipitation (MeRIP) procedure was performed according to instructions issued by the manufacturer using a Magna MeRIP™ m6A kit (#17–10,499, Merck Millipore, MA). Briefly, purified mRNA was digested by DNase I and then fragmented into ∼100 nt using RNA fragmentation reagent and incubated at 94 °C. After fragmenting, the stop buffer was added, following which standard ethanol precipitation was performed and collected. The anti-m6A antibody for 12 μg was pre-incubated with 50 μl beads in IP buffer (150 mM NaCl, 0.1% NP-40, 10 mM Tris–HCl, pH 7.4) at room temperature for 1 h. Next, 6 μg of fragment mRNAs were added to the antibody-beads mixture and incubated at 4 °C for 4 h on a rotator. After adequate washing, immunoprecipitated mixture was digested using high concentration of proteinase K, and the bound RNAs were extracted using phenol-chloroform method and ethanol precipitation and were used for qPCR analysis. qPCR analysis determined the modification of m6A in PTEN analysis based on precise primers. All m6A sites of PTEN were predicted using SRAMP (http://www.cuilab.cn/sramp). We created primers to make sure that the target sequence included all these sites within 100 nt length.

### Intracerebroventricular injection

Rats were anesthetized with 3% isoflurane, secured on a stereotaxic apparatus (Narashige, Tokyo, Japan), and a midline incision was made to expose the skull for brain coordinates. A burr hole was drilled and, using a Hamilton syringe, we then stereotactically implanted 1 million cells at a rate of 0.3 μl/ min into the left lateral cerebral ventricle at an injection depth of 2.5 mm at a point 1.5 mm lateral to the midline and 0.8 mm posterior from bregma. Following the injection, the needle was kept in place for 5 min to facilitate virus diffusion, then slowly withdrawn. Finally, guide holes were closed using bone wax, sutured, and disinfected.

### Transmission electron microscopy

As a preliminary step for transmission electron microscopy (TEM) analysis, samples were fixed with 2.5% glutaraldehyde for 1 h at room temperature, and dehydrated in ethanol for 10 min. In addition to dehydrating the sample and embedding it in epoxy resin, sections were cut and stained with uranyl acetate and lead citrate after dehydration. The microstructure of hippocampus was characterized by TEM (JEM-2100). Using a TEM, we observed autophagy and autolysosome ultrastructure. The ultrastructural morphological changes of synapses were also characterized by TEM.

### Golgi staining

Rats were sacrificed by rapid decapitation and their brains were removed for Golgi-Cox staining. Dissected brains were immersed in 5% potassium dichromate, 5% mercuric chloride, and 5% potassium chromate for 35 days in Golgi-Cox solution (FD, NeuroTechnologies Columbia, Canada). A freezing microtome was used to section the brains coronally at 150 μm after they had been placed in 20% sucrose solution overnight. Staining was followed by two rinses in distilled water, a 2-min treatment with 5% sodium thiosulfate, and two rinses in distilled water. These were dehydrated by successive dips in 70%, 90%, and 100% ethanol and xylene followed by three rinses with bidistilled water. Images were captured using a Nikon E600 Camera.

### Morris water maze test

Memory and spatial learning can be studied using the Morris water maze (MWM) test, with which rats were assessed 21 days post-HI. The MWM consists of a circular pool (120 cm diameter) filled with water (21–22 °C). There are four made quadrants, one of which contains a hidden platform (10 cm diameter) submerged 4 cm below the surface. Over repeated trials, rats are able to learn how to find the escape platform using visual clues on the maze wall. The maximum time to find the platform was set at 60 s for each trial. For the acquisition phase, all rats were tested four times daily for five consecutive days, starting from a different quadrant each day. A probe trial without the platform was conducted on the sixth day to examine spatial memory. Videos were recorded by and analyzed using Ethovision 11.0.

### Statistical analysis

SPSS Statistics 21.0 (IBM, Armonk, USA) was used for analyses. Data are presented as means ± standard deviations. To compare normally distributed data of groups, a *t*-test or one-way ANOVA was used. A Shapiro Wilk normality test was performed to evaluate the normality of the data’s distribution. Tukey’s test was conducted if the data were normally distributed. When they were not normally distributed, a Kruskal-Wallis test was conducted. An analysis of the Morris water maze training data was performed using two-way repeated-measures ANOVA and Tukey’s test. We considered P values < 0.05 to be statistically significant.

## Results

### Increased m6A RNA modification in the hippocampus of HI neonatal rats

**Fig. 1:**
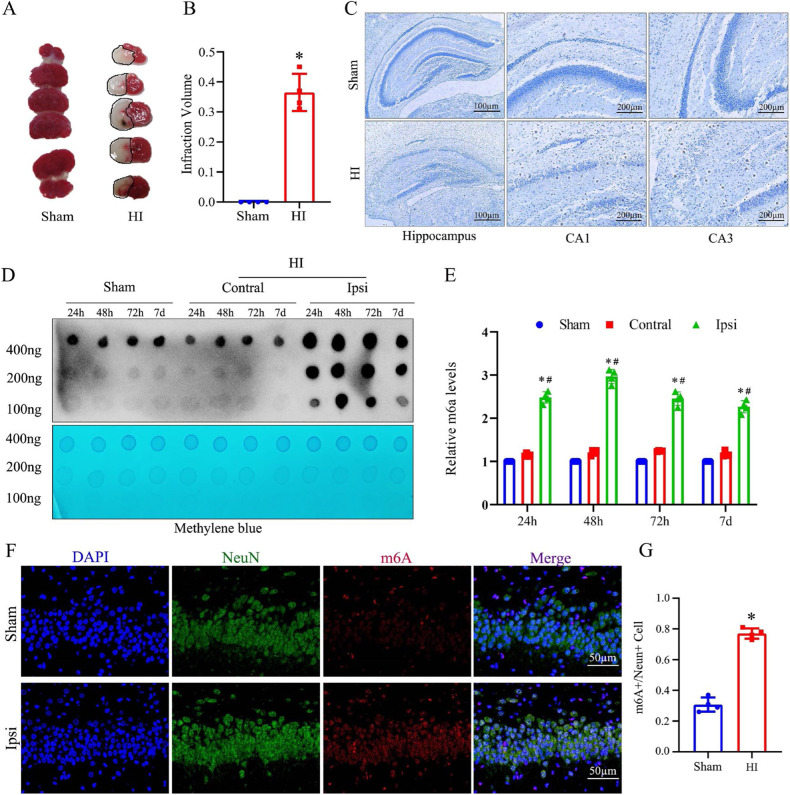
HI contributes to neuronal impairment and augmentation of hippocampal global methylation. Neonatal rats were treated with HI exposure at PND7. **A**, **B** TTC staining was used to quantify cerebral infarct volumes in rats from different groups 48 h post-HI. **C** Nissl staining was used to assess relative changes and examine neuronal cell death in hippocampal neurons (scale bar = 100/200 µm). **D**, **E** Hippocampal m6A levels were analyzed by dot blot. m6A quantification is shown in the right panel. **F**, **G** Immunofluorescence was conducted to evaluate m6A in sham and HI samples (scale bar = 50 µm). Data are presented as means ± standard deviations. *p < 0.05 vs. the Sham group; #*p* < 0.05 vs. the Contral group.

We first evaluated the infarct volumes post-HI using TTC staining. Following HI injury, there was a significant increase in brain infarction size compared to the sham group (Fig. 1A, B). Nissl staining of brain sections was then performed for a more detailed tissue analysis. Hippocampal neurons in the HI group had fewer Nissl bodies, lighter staining, irregular cell arrangement in the CA1 region, and blurred nuclei (Fig. 1C). The mammalian hippocampus, a cortical region involved in mnemonics and spatial behavior, is particularly sensitive to hypoxia. As described above, we observed distinct signs of severe neuronal damage in the hippocampal neurons of rats subjected to HI injury. To determine whether m6A methylation plays a functional role in HIBD, we examined global m6A in the hippocampi of HI rats compared with normal control samples. Then, we performed a dot blot and immunofluorescence to determine global m6A RNA methylation (Fig. 1D–G). The results showed an overall increase in m6A post-HI. It is noteworthy that global m6A RNA methylation increased steadily post-HI, and was most obvious 48 h post-HI. Thus, we aimed to further investigate the mechanisms by which m6A RNA is modified in HIBD.

### Decreased FTO expression in hippocampi of HI neonatal rats

**Fig. 2:**
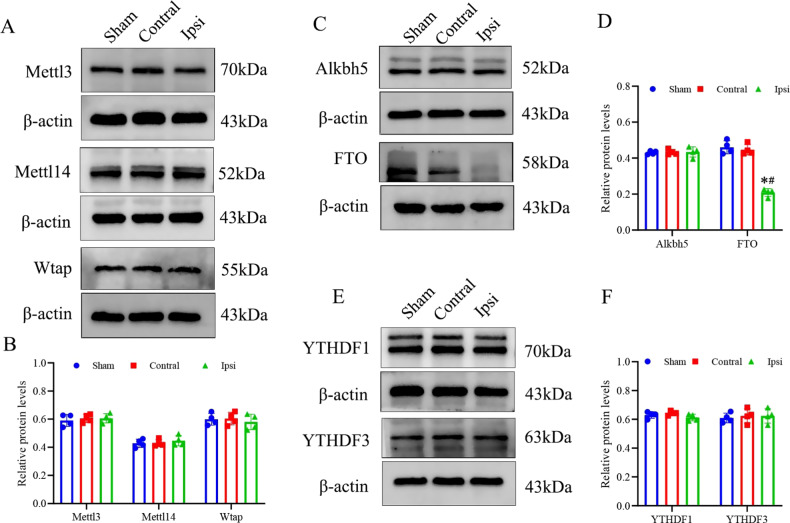
Hippocampal FTO protein was decreased in HI-induced brain damage in neonatal rats. Western blot analysis of methylase complex Mettl3, Mettl14, Wtap (**A**, **B**), major demethylase Alkbh5 and FTO (**C**, **D**) YTH domain contain protein YTHDF1, YTHDF3 (**E**, **F**) among sham, contralateral and ipsilateral hippocampus samples after HI exposure. β-Actin was used as the loading control. Blots shown are representative of at least four experiments with similar results. Data are presented as means ± standard deviations. *p < 0.05 vs. the Sham group; #*p* < 0.05 vs. the Contral group.

Researchers have identified key regulatory enzymes and proteins involved in m6A modification, including methyltransferases, demethylases, and binding proteins [36]. As a precursor to our m6A modification analysis, we examined the expression levels of methyltransferase complexes Mettl3, Mettl14, and Wtap, demethylases Alkbh5 and FTO, and binding proteins including YTHDF1 and YTHDF3. HI significantly elevated m6A RNA modification level, further western blot data revealed that FTO protein expression was significantly decreased in the HI group compared with sham controls (Fig. 2C, D). HI did not demonstrate any noticeable impact on other m6A regulators, including m6A methyltransferase proteins (Mettl14, Wtap, and Mettl3), binding proteins (YTHDF1 and YTHDF3), and m6A demethylase Alkbh5 (Fig. 2).

Collectively, these data presented in this study support the notion that the loss of FTO could serve as a significant molecular characteristic, potentially elucidating the observed elevation in m6A RNA modification levels within post-HI hippocampi. FTO, a demethylase involved in the removal of the m6A modification from mRNA, has established roles in normal central nervous system functions and the pathobiology of neurological disorders [37, 38]. Based on these findings, we formulated a hypothesis suggesting the involvement of FTO in the pathophysiology of HIBD.

### Differentially m6A-modified transcripts are linked to a number of pathological processes in hippocampi of HI neonatal rats

**Fig. 3:**
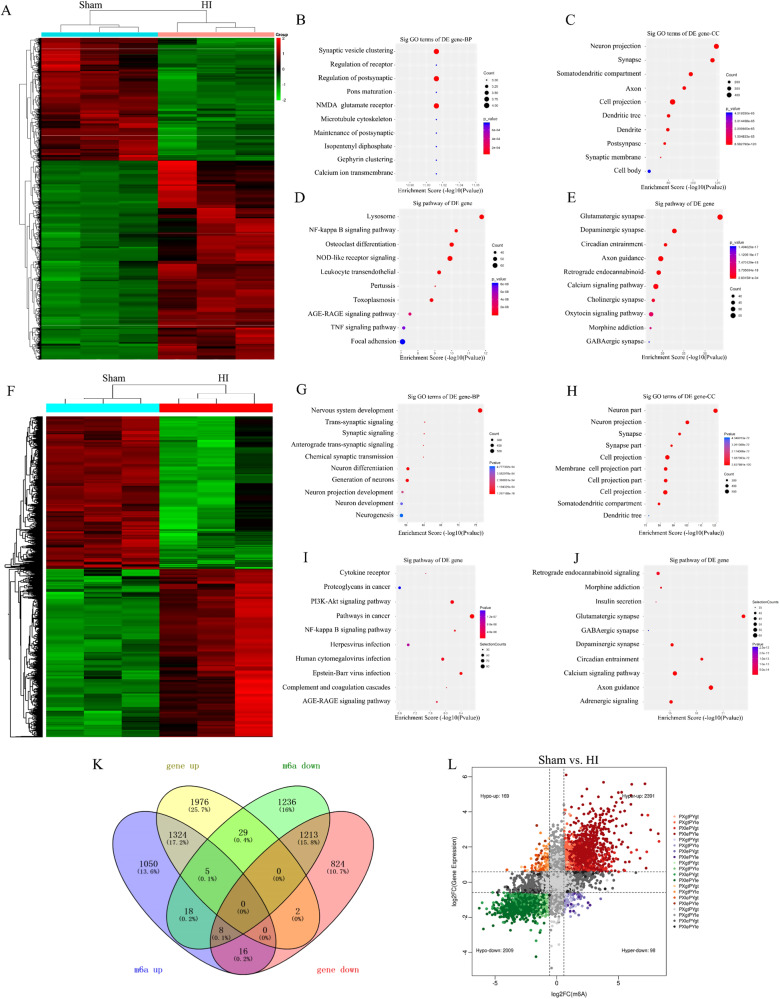
Conjoint analysis of hippocampal RNA-seq and methylated RNA immunoprecipitation sequencing 2 days post-HI. **A** Cluster heat map shows differentially expressed RNA between the sham and HI treatment groups (*n* = 3). **B**–**E** GO and KEGG analysis were performed using genes that were significantly differentially expressed in the HI treatment group (Edge R *P* ≤ 0.05). Significantly enriched biological processes (BP), molecular function (MF) and cellular component (CC) are depicted in the RNA-seq graph. **F** Cluster heat map shows differentially methylated RNA between the sham and HI treatment groups (*n* = 3). **G**–**J** GO and KEGG analysis were performed using genes that were significantly differentially methylated in the HI treatment group (Edge R *P* ≤ 0.05). **K** Venn diagrams of aberrantly methylated differentially expressed genes**. L** Four quadrant graph showing the distribution of transcripts with significant changes in both m6A-modified level and corresponding mRNA expression 48 h post-HI.

To assess the association between m6A modification and mRNA expression in HIBD, we conducted a comprehensive and sequential analysis that integrated m6A RNA microarray and RNA sequencing (RNA-seq) datasets. This approach allowed us to investigate the connections between m6A modification and mRNA expression. Subsequently, through RNA-seq analysis, we observed distinct separation and clustering patterns among all the examined groups (Fig. 3A). The results obtained from the RNA-seq analysis revealed that the differentially expressed genes (DEGs) were significantly enriched in various synapses or synaptic components, as indicated by both the KEGG pathway enrichment and GO enrichment analyses. Moreover, the downregulated targets displayed a marked overrepresentation of GO terms related to nervous system development. These findings strongly suggest the involvement of these genes in synaptic processes and neuronal development (Fig. 3B–E). Consequently, exposure to HI conditions led to the depletion of genes associated with synapses, and the DEGs were primarily enriched in various synapses, indicating a reduction in synaptic connections within the hippocampi of neonatal rats following HI injury. Furthermore, the analysis highlighted the enrichment of methylated genes under GO terms related to neuron generation, trans-synaptic signaling, and response to stimuli. These findings suggest that the HI insult impacts synaptic function and neuronal communication, highlighting the potential role of m6A modifications in these processes (Fig. 3F, J). A combination analysis of m6A RNA microarray and RNA-seq data identified DEGs that were found to be enriched in various synaptic pathways. Notably, genes associated with synapses exhibited downregulation and differential m6A modification in the hippocampi of neonatal rats with HI injury (Fig. 3K, L). By integrating the RNA-seq analysis with the m6A microarray analysis, we observed that transcripts with m6A modifications exhibited higher expression levels compared to those without m6A modifications. This finding suggests that m6A modification may play a regulatory role in maintaining gene expression homeostasis in the hippocampi of neonatal rats with HI injury.

### Deregulation of AKT pathway and activation of autophagy in hippocampi of HI neonatal rats

**Fig. 4:**
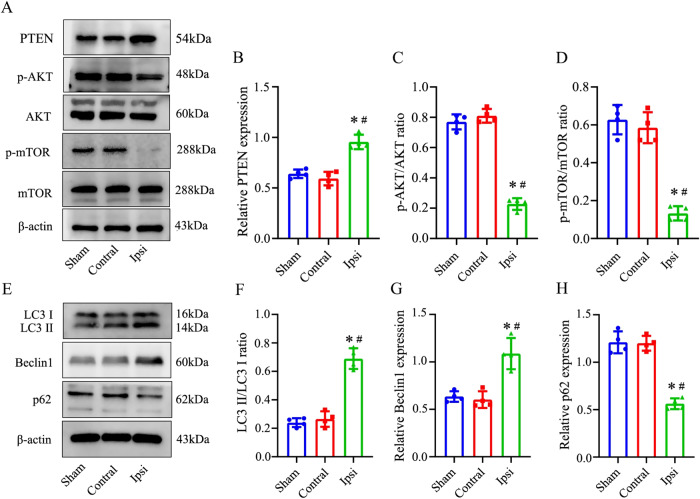
Activation of AKT pathway and augment of autophagic flux induced by HI treatment. **A**–**D** Western blot analysis of expression of PTEN and AKT, mTOR phosphorylation among sham, contralateral and ipsilateral hippocampus samples at 48 h after HI exposure. **E**–**H** Western blot analysis for LC3II and p62, Beclin1 after HI exposure in neonatal rats. The blots shown are representative of at least 4 experiments with similar results. Data are presented as means ± standard deviations. *p < 0.05 vs. the Sham group; #*p* < 0.05 vs. the Contral group.

Considering the crucial involvement of the AKT pathway in autophagy, it is noteworthy that its activation is enhanced in the striatum following neonatal cerebral HI [39]. However, the initiation of excessive autophagy may be associated with neuronal damage [40]. Recognizing the pivotal role of the AKT pathway in controlling autophagy during HIBD, we conducted an evaluation of the protein expression and phosphorylation levels of key signaling molecules in the AKT pathway. A decrease in the levels of phosphorylated AKT and mTOR was observed, along with an increase in the expression of PTEN, which acts as a negative regulator in the Akt pathway (Fig. 4A–D). These findings suggest that the AKT/mTOR signaling pathway is suppressed in response to neonatal HIBD. Further, post-HI increased expressions of autophagic proteins LC3II and Beclin1, and reduced expression of p62 were typically associated with the accumulation of autophagy (Fig. 4E–H). The cumulative evidence presented demonstrates the induction of suppressed AKT signaling and enhanced autophagic flux in response to HI.

### FTO is involved in aberrant m6A modification in hippocampi of HI neonatal rats

**Fig. 5:**
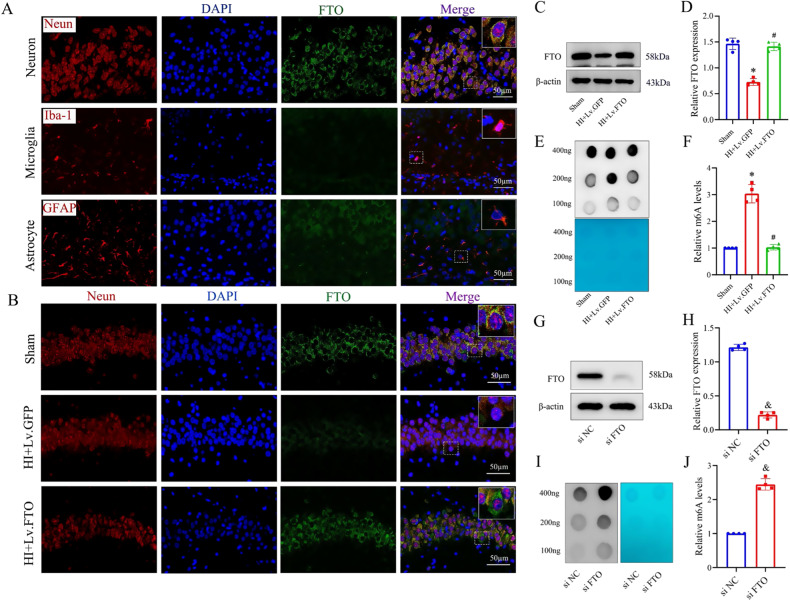
FTO is involved in aberrant m6A modification in hippocampi of HI neonatal rats. **A** Representative immunofluorescence images of FTO (green), Neun (red) and GFAP (red) and Iba1 (red) staining in sections of the hippocampus after HI exposure. **B** Immunostaining was used to evaluate the FTO level in hippocampal overexpression. **C**, **D** FTO levels in the ipsilateral hippocampus were measured by western blot. **E**, **F** Hippocampal m6A levels in sham, HI+Lv.GFP, and HI+Lv.FTO groups were analyzed by m6A dot blot. m6A quantification is shown in the right panel. Scale bar is 50 µm. *n* = 4 animals per group. Data are presented as means ± standard deviations. **G**, **H** FTO protein levels were measured by western blot in primary cultured hippocampal neurons after FTO knockdown. **I**, **J** m6A levels between groups were analyzed by m6A dot blot. m6A quantification is shown in the right panel. *p < 0.05 vs. the Sham group; #*p* < 0.05 vs. the HI+Lv.GFP group; & *p* < 0.05 vs. the si NC group.

Internal modifications of m6A are reversible and can be enzymatically removed by proteins known as m6A demethylases. We conducted additional investigations to explore the involvement of the m6A demethylase FTO in the regulation of m6A hypermethylation in the hippocampi of neonatal rats with HIBD. In immunofluorescence staining analyses of GFAP-positive astrocytes, Iba-1-positive microglia, and Neun-positive neurons, FTO exhibited predominant expression in neurons rather than astrocytes and microglia (Fig. 5A). Similarly, double immunofluorescence staining demonstrated a significant decrease in FTO expression in neurons of HIBD rats (Fig. 5B). To explore the involvement of FTO in the hippocampus and assess its role as a direct regulator of m6A, we utilized lentiviral vectors (Lv.) as a transgenic tool for targeted overexpression of FTO in primary hippocampal neurons and the hippocampus (Fig. 5C, D). Interestingly, the overexpression of FTO in primary hippocampal neurons cultured under OGD treatment reversed the aberrant increase in m6A modification in RNA induced by OGD (Fig. 5E, F). These findings suggest that FTO plays a critical role as a key regulator of m6A modification in primary hippocampal neurons. To enhance the robustness of our findings, we conducted an examination of the m6A levels in primary cultured hippocampal neurons where FTO expression was knocked down. Our analysis revealed a notable increase in the m6A modification levels within the cultured cells exhibiting FTO knockdown (Fig. 5G–J). Additionally, the expression of other m6A modification enzymes, including methylase complex Mettl3, Mettl14, Wtap, major demethylase Alkbh5 and FTO, YTH domain contain protein YTHDF1, YTHDF3 were not affected after FTO overexpression (Fig. S1).

### FTO-dependent m6A demethylation destabilized PTEN mRNA and reduced autophagy in hippocampi of HI neonatal rats

**Fig. 6:**
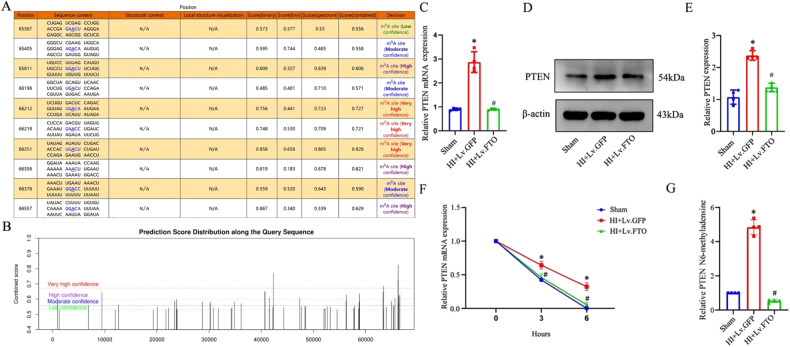
Increased FTO expression destabilizes PTEN mRNA in neonatal rats after HI. **A**, **B** Predicted m6A site in PTEN mRNA of a sequence-based m6A modification site predictor (SRAMP). **C** After OGD-R treatment, primary hippocampal neurons were harvested for real-time quantitative polymerase chain reaction (RT-qPCR) to assess PTEN expression, and loading control GAPDH. **D**, **E** PTEN levels in the ipsilateral hippocampus were measured by western blot. **F** PTEN mRNA half-life after FTO overexpression measured by incubating cells with actinomycin D, extracting total RNA at the times shown, and measuring PTEN and (housekeeping) GAPDH mRNA levels by RT-qPCR analysis. **G** The relative levels of m6A in PTEN mRNA were tested by MeRIP-qPCR from primary cultured hippocampal neurons with overexpression of FTO. *p < 0.05 vs. the Sham group; #*p* < 0.05 vs. the HI+Lv.GFP group.

**Fig. 7:**
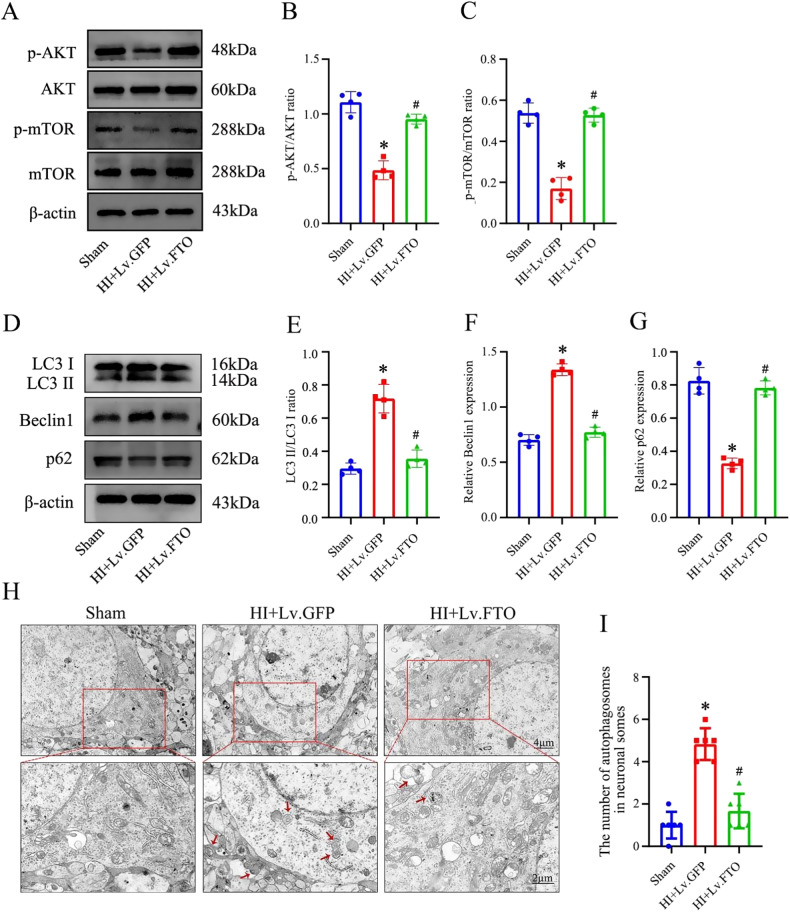
Hippocampal overexpression of FTO contributes to AKT pathway activation after HI in neonatal rats. **A**–**C** After FTO overexpression, AKT signaling pathway protein levels were upregulated and activated. **D**–**G** Total hippocampal sample LC3II, p62, Beclin1, and b-actin levels in each group were analyzed by western blot. Representative bands are shown. At least four independent experiments were carried out. **H**, **I** Transmission electron microscopy was used to analyze the formation of autophagosomes in the hippocampi of each group. Scale bars used in zoomed-out and zoomed-in images are shown. Data are presented as means ± standard deviations. *p < 0.05 vs. the Sham group; #*p* < 0.05 vs. the HI+Lv.GFP group.

We observed that HI caused inhibition of the AKT signaling pathway and promoted enhanced autophagy. PTEN positively regulates autophagy by inhibiting AKT activity. Given that FTO is a demethylase, we hypothesized that FTO modulates autophagy through an m6A-dependent pathway. Given that m6A modification has an impact on gene expression, we hypothesized that m6A modification of PTEN mRNA might regulate its expression. To investigate this, we utilized SRAMP (Site-Specific RNA Demethylation and Methylation Prediction) to predict m6A sites. Interestingly, we identified numerous m6A sites with high confidence predominantly located in the 3’ untranslated region (3’ UTR) of PTEN mRNA (Fig. 6A, B). The overexpression of FTO reversed the OGD/R-induced increase in both PTEN RNA and protein levels (Fig. 6C–E). The overexpression of FTO led to a decrease in PTEN mRNA stability, characterized by an increased rate of mRNA decay (Fig. 6F). Furthermore, we observed a noticeable increase in m6A-modified PTEN mRNA in primary cultured hippocampal neurons following OGD/R, as assessed by the MeRIP-qPCR assay. Importantly, this alteration was significantly reversed upon overexpression of FTO (Fig. 6G). The aforementioned findings suggest a potential role of FTO in destabilizing PTEN mRNA in an m6A-dependent manner. Therefore, we further explored the impact of FTO overexpression on the AKT signaling pathway and autophagy following HI and OGD/R treatment. To achieve FTO overexpression, a lentiviral overexpression vector was utilized. Western blots were used to evaluate the protein and phosphorylation levels of the AKT pathway and several autophagy-related markers, including LC3II, Beclin1, and p62. Phosphorylation of AKT, and mTOR were increased in the HI+Lv.FTO group compared with the HI+Lv.GFP group (Fig. 7A–C). In addition, FTO overexpression led to increased expression of p62 and reduced LC3II and Beclin1 expressions, suggesting a negative correlation between FTO and autophagy (Fig. 7D–G). TEM was utilized to investigate autophagosomes, revealing that hippocampi overexpressing FTO showed a decreased accumulation of autophagic vesicles (Fig. 7H, I). Based on these findings, we formulated the hypothesis that FTO regulates the expression of PTEN by modulating the degradation of PTEN mRNA. Furthermore, we propose that the overexpression of FTO in the hippocampus leads to the suppression of autophagic flux, primarily through the downregulation of PTEN, which subsequently activates the AKT pathway.

### Hippocampal FTO overexpression attenuates synaptic and cognitive impairment of HI neonatal rats

**Fig. 8:**
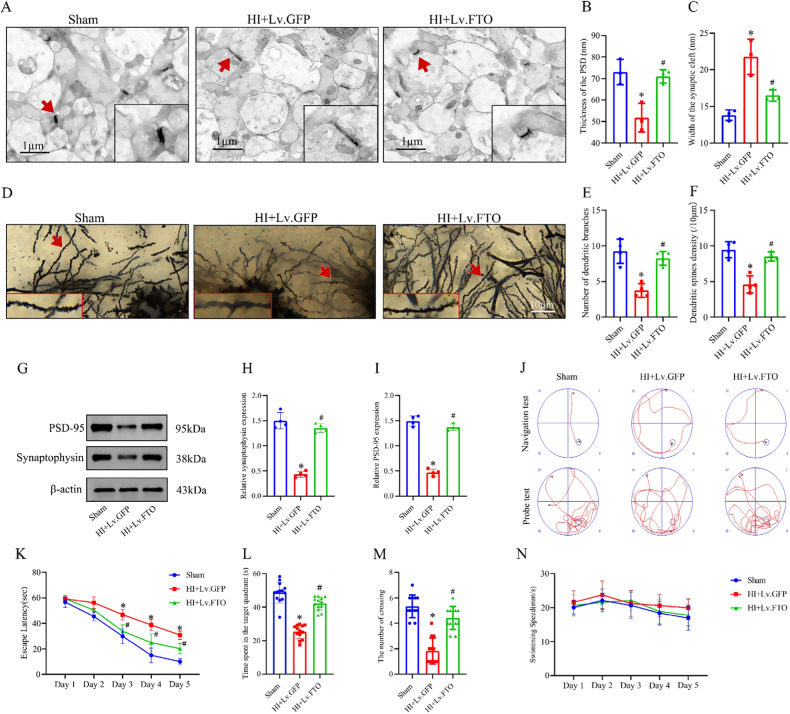
Hippocampal overexpression of FTO involved in synaptic and cognitive function improvements. **A**–**C** Synaptic ultrastructure changes in the hippocampal CA1 region were observed under TEM for neonatal rats in each group. Representative high magnification photomicrograph shows differences in PSD thickness and synaptic cleft width among the three groups (red arrows indicate synaptic linkage). Scale bar = 1 µm. **D**–**F** Dendritic complexity of Golgi-stained neurons shown by representative images of hippocampal CA1 neurons in the three neonatal rat groups 48 h post-HI. Quantification of dendritic spines’ branches and density (*n* = 4 per condition) are shown. **G**–**I** Hippocampal PSD95 expression and synaptophysin were measured by western blot 48 h post-HI. **J**–**N** Representative swimming tracks (red routes) of the rats from the place navigation test on day 5 and the probe test on day 6. Escape latency, time spent in the target quadrant, number of platform crossings, and swim speed were evaluated to measure spatial learning and memory post-HI. Data are presented as means ± standard deviations. *p < 0.05 vs. the Sham group; #*p* < 0.05 vs. the HI+Lv.GFP group.

Synapses serve as the primary sites of neuronal damage, and the progressive dysfunction of synapses is strongly associated with cognitive decline. Previous research has demonstrated that autophagy dysregulation is implicated in synaptic dysfunction, cognitive impairments, and neuroinflammation induced by HI [41, 42]. In our study, we aimed to delve deeper into the impact of FTO on impaired synaptic integrity and cognitive function in the context of HI. The synaptic ultrastructure in the hippocampus was examined using TEM. In the HI group, notable pathological changes were observed, including a significant reduction in postsynaptic density (PSD) thickness and widening of the synaptic cleft compared to the sham group. Remarkably, in the FTO overexpression group, improvements in synaptic ultrastructure impairments in the hippocampus were observed compared to the HI group (Fig. 8A–C). Additionally, Golgi-Cox staining was employed to investigate the impact of FTO in rats with HIBD on the neuronal morphology specifically in the CA1 region of the hippocampus. In pyramidal neurons, we assessed the density of dendritic spines along individual dendrites. Our findings revealed a significant reduction in the density of dendritic spines, spine branches, and dendritic length in CA1 pyramidal neurons stained with Golgi technique in HIBD rats. Compared to the HI group, FTO overexpression significantly enhanced dendritic complexity in the CA1 region of the hippocampus. This enhancement was observed in terms of increased spine branches, dendritic length, and spine density (Fig. 8D–F). In neurons treated with HI, the expression of synaptic markers PSD95 and synaptophysin was found to be decreased compared to the sham group. However, the reduction in expression could be effectively reversed by overexpressing FTO (Fig. 8G–I). Based on our findings, we postulate that the overexpression of FTO has the potential to alleviate the HI-induced abnormalities in synaptic structure and function.

To evaluate learning and memory behaviors, MWM tests were conducted as a subsequent step. A rapid decrease in escape latency was seen in all groups over the course of the five navigation test days, and no significant interaction was found between training days and groups, suggesting an improvement in spatial learning and memory over time. No significant differences in escape latency between the groups were observed on day 1. However, starting from day 2 until day 5, the HI+Lv.GFP group exhibited significantly longer escape latencies compared to the sham group. Interestingly, HI+Lv.FTO group rats had a shorter escape latency from days 2–5 than did the HI+Lv.GFP group, indicating cognitive improvements (Fig. 8K). Similarly, the HI+Lv.FTO group exhibited a longer duration spent in the target quadrant and a greater number of platform crossings during the probe trial (Fig. 8J, L, M). There were no significant differences in swim speed among the groups, indicating that the observed variations in the outcomes of the MWM test were likely attributable to cognitive changes rather than differences in physical abilities or swimming speed (Fig. 8N). These results suggest that HI impaired synaptic and cognitive function, whereas FTO overexpression had neuroprotective effects in HIBD.

## Discussion

Neuroepigenetics is emerging as an independent field within neuroscience, enhancing the study of one of nature’s most complex systems [43–45]. Epigenetics is increasingly appreciated to play critical roles in neural development and diseases, and particularly m6A methylation [46, 47]. Herein, we explored the regulatory role and underlying mechanism of FTO in the synaptic and cognitive function in neonatal rats after HI. Our main findings include the following: (1) HI-induction in neonatal rats increased m6a methylation and reduced FTO expression; (2) FTO overexpression destabilized PTEN mRNA to modulate autophagy post-HI in neonatal rat hippocampi; and (3) hippocampal FTO overexpression attenuates HI-induced synaptic loss and cognitive impairment in neonatal rats. Our findings indicate that FTO may play a key role in HI-induced synaptic and cognitive impairment through regulation of autophagy.

Dysregulation of m6A methylation is strongly associated with neurological diseases, including traumatic brain injury, Alzheimer’s disease, Parkinson’s disease, and major depressive disorder [48, 49]. However, the mechanism underlying RNA m6A methylation that is responsible for neonatal HIBD pathology is incompletely understood. The dynamic expression patterns of writer, reader, and eraser proteins complicate our understanding of the precise functional consequences of aberrant modification deposition on RNA. To understand the general changes in these m6A-related regulators during HIBD, we estimated their signatures in sham and HIBD hippocampal tissue. We observed global augmentation in RNA m6A methylation, which reached its highest level on day 2 post-HI. Reduced demethylase FTO expression was observed post-HI, which led to increased methylation in HIBD. However, hippocampal FTO overexpression could reverse HI-induced hypermethylation. Remarkably, we observed a significant increase in global RNA methylation levels in primary cultured hippocampal neurons with FTO knockdown. Additionally, the overexpression of FTO in the hippocampi of HI neonatal rats did not have a significant impact on the expression of critical enzymes involved in m6A RNA modification. Thus, we proposed that FTO is a key regulator of m6A modification in hippocampal neurons of neonatal rats post-HI.

HI leads to synaptic deficits, especially in hypoxia-sensitive brain areas like the hippocampus [50, 51]. Major functions of the hippocampal system, cognition and memory, depend on synaptic transmission properties, while cognitive symptom severity is significantly correlated with hippocampal synapse loss [52]. Several studies have linked autophagy to brain injury in the context of neurodegenerative diseases [53, 54]. In the presence of HI, autophagy degrades unwanted proteins, while abnormal activation of autophagy may promote cell death [16]. Numerous studies have explained that aggravated autophagy accumulation eventually results in synapse and cognitive impairment, as is seen in stroke [15, 55]. Treatment with the mTOR inhibitor rapamycin mitigates hippocampal neuron death in neonatal rats post-HI [56]. Consistent with this, we observed HI-induced increased LC3II and autophagy accumulation in neonatal rats, which also performed worse on memory and global cognition tasks. In general agreement with our findings, hippocampal slice of HI-exposed neonatal rat brains exhibit increased autophagosome formation, with a negative correlation reported between autophagy and synaptic function [56, 57]. A previous study reported that excessive autophagic activation induces autophagic programmed cell death, and inhibition of autophagy partially improves spatial learning and memory in an immature brain HIBD model [41]. Based on these findings, we hypothesized that the mechanisms underlying HIBD-induced cognitive dysfunction are related to hippocampal autophagy. Thus, we then investigated the mechanisms of m6A in hippocampal autophagy in HI neonatal rats.

Based on the overlap of m6A profiling data and RNA-seq in HIBD models, we observed that m6A-tagged transcripts were expressed more highly than were non-m6A transcripts at steady state. KEGG pathway analysis of strongly expressed genes with upregulated m6A methylation was used to determine the effect of m6A in HIBD. KEGG analysis revealed that the AKT pathway is significantly enriched in hypermethylated genes, suggesting its involvement in the pathological process in a m6A-dependent manner. A recent study showed that FTO positively regulates the proliferation and differentiation of 3T3-L1 cells via enhanced AKT signaling, indicating that FTO may inhibit autophagy. Our results concur with previous research in that FTO overexpression strongly upregulated the hippocampal AKT pathway post-HI. To further explain the molecular mechanisms by which m6A regulators are involved in HIBD, we examined the correlation between FTO expression and autophagy flux.

PTEN is a classical tumor suppressor that negatively regulates AKT/mTOR signaling, and positively regulates autophagy gene expressions [58]. Herein, we validated PTEN as a direct target of FTO in HI-exposed hippocampus. The m6A demethylase FTO was found to be the first to play an important role in regulating RNA stability, translation, and interactions with other molecules [59]. As a positive autophagy regulator, PTEN mRNA contains numerous m6A sites with high confidence, suggesting that methylation modification affects some PTEN expression. The data herein indicate that HI elevates PTEN expression and suppresses the AKT signaling pathway, and that it enhances autophagy in the hippocampus of neonatal rats. Another group has also shown that PTEN expression is reduced due to m6A mRNA instability caused by demethylase ALKBH5 in cadmium-transformed cells [60]. Consistently, we found that FTO overexpression negatively affects PTEN expression in primary cells via destabilize PTEN mRNA. Further study found that FTO overexpression could decrease the m6A levels of PTEN mRNA in primary cells, suggesting that FTO regulates PTEN expression in an m6A-dependent manner. However, we then observed a strong downregulation of autophagic-related protein and reduced autophagosome formation in FTO overexpression HIBD rats. Accordingly, we proposed that FTO overexpression inhibits autophagy, partially via destabilization of PTEN mRNA under HI conditions.

Autophagy has been shown to regulate synaptic function post-HI. Herein, we examined learning and memory in sham, HI, and FTO overexpression group rats using the MWM test. These results showed that cognitive impairment is induced by HI. Furthermore, FTO overexpression rats exhibit improvement in learning and memory function post-HI. Importantly, our study revealed that FTO overexpression restored synaptic marker protein levels concomitant with improved synaptic loss and spine maturation, indicating amelioration of synaptic integrity. These molecular results were consistent with the behavioral findings, suggesting that FTO-m6A function reduced autophagy post-HI through destabilized PTEN mRNA, which is involved in synaptic and cognitive improvements. Our findings demonstrate that the FTO/PTEN axis acts as a critical regulator in HI neonatal rats, linking synaptic and cognitive impairment to autophagy.

This study was not without several limitations. First, m6A methylation site prediction and functional verification should be performed at multiple levels to confirm the FTO-dependent epigenetic regulatory mechanism of PTEN. Second, we specifically focused on the role of FTO in HIBD; in future research, we will use specific FTO inhibitors to demonstrate that reduced FTO expression may contribute to accentuated neuronal injury post-HI. Ultimately, these are important questions that should be addressed by future studies, especially in rescue studies, as the roles of FTO and PTEN in HIBD require further clarification.

**Fig. 9:**
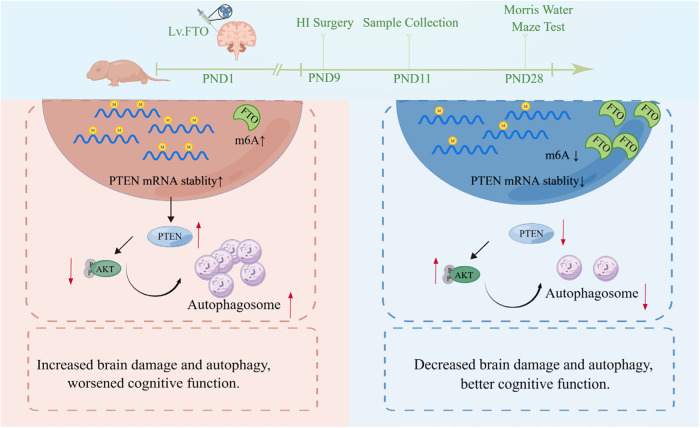
Schematic diagram illustrates the mechanism of FTO upregulated in a m6A-dependent manner to ameliorates synaptic and cognitive impairment and inhibiting autophagy in HIBD.

Collectively, the present study offers compelling evidence that the aberrant m6A demethylase FTO contributes to synaptic and cognitive impairment in neonatal HIBD by activating Akt/mTOR-mediated autophagy through the destabilization of PTEN mRNA. Therefore, targeting FTO emerges as a promising strategy for future neuroprotective treatments in neonatal HIBD (Fig. 9).

### Supplementary information

aj-checklist

Figure Supplement 1

F2 western blot original data

F4 western blot original data

F5 western blot original data

F6 western blot original data

F7 western blot original data

F8 western blot original data

FS1 western blot original data

## Data Availability

The original contributions presented in the study are included in the article/Supplementary Material; further inquiries can be directed to the corresponding author.
