# Supplementary material for: N6-methyladenosine demethylase FTO regulates synaptic and cognitive impairment by destabilizing PTEN mRNA in hypoxic-ischemic neonatal rats
Source: Cell Death Dis. 2023 Dec 13;14(12):820. doi: 10.1038/s41419-023-06343-5 (PMC10719319; doi:10.1038/s41419-023-06343-5)
Supplement: Supplementary file 2 — Figure Supplement 1 [file 41419_2023_6343_MOESM2_ESM.pdf]

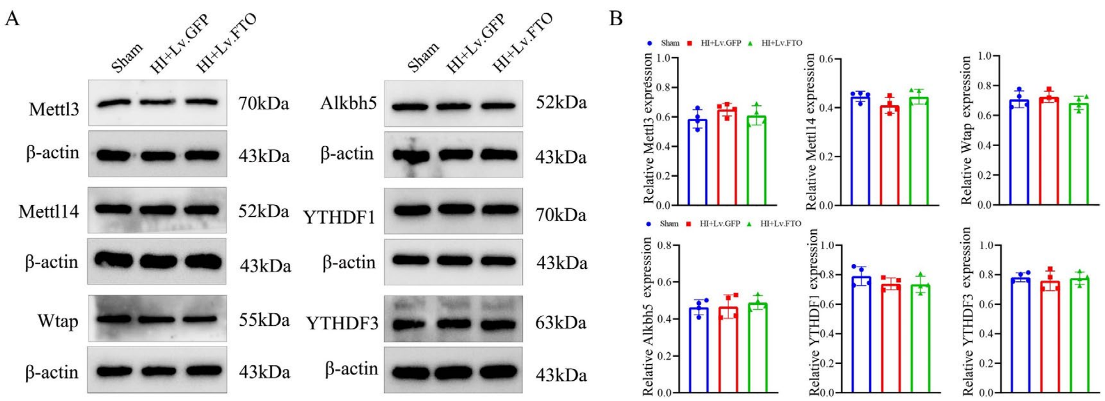

Fig. S1 Expression patterns of major methyltransferases and demethyltransferase, YTH domain protein after FTO overexpression in in hippocampi of HI neonatal rats

(A-B) Western blot analysis of methylase complex Mettl3, Mettl14, Wtap, demethylase Alkbh5, YTH domain contain protein YTHDF1, YTHDF3 among sham, HI+Lv.GFP and HI+Lv.FTO groups. β-Actin was used as the loading control. Blots shown are representative of at least 4 experiments with similar results. Data are presented as means ± standard deviations. * $p < 0.05$ vs. the Sham group; # $p < 0.05$ vs. the HI+Lv.GFP group.
