# Supplementary material for: N6-methyladenosine demethylase FTO regulates synaptic and cognitive impairment by destabilizing PTEN mRNA in hypoxic-ischemic neonatal rats
Source: Cell Death Dis. 2023 Dec 13;14(12):820. doi: 10.1038/s41419-023-06343-5 (PMC10719319; doi:10.1038/s41419-023-06343-5)
Supplement: Supplementary file 3 — F2 western blot original data [file 41419_2023_6343_MOESM3_ESM.pdf]

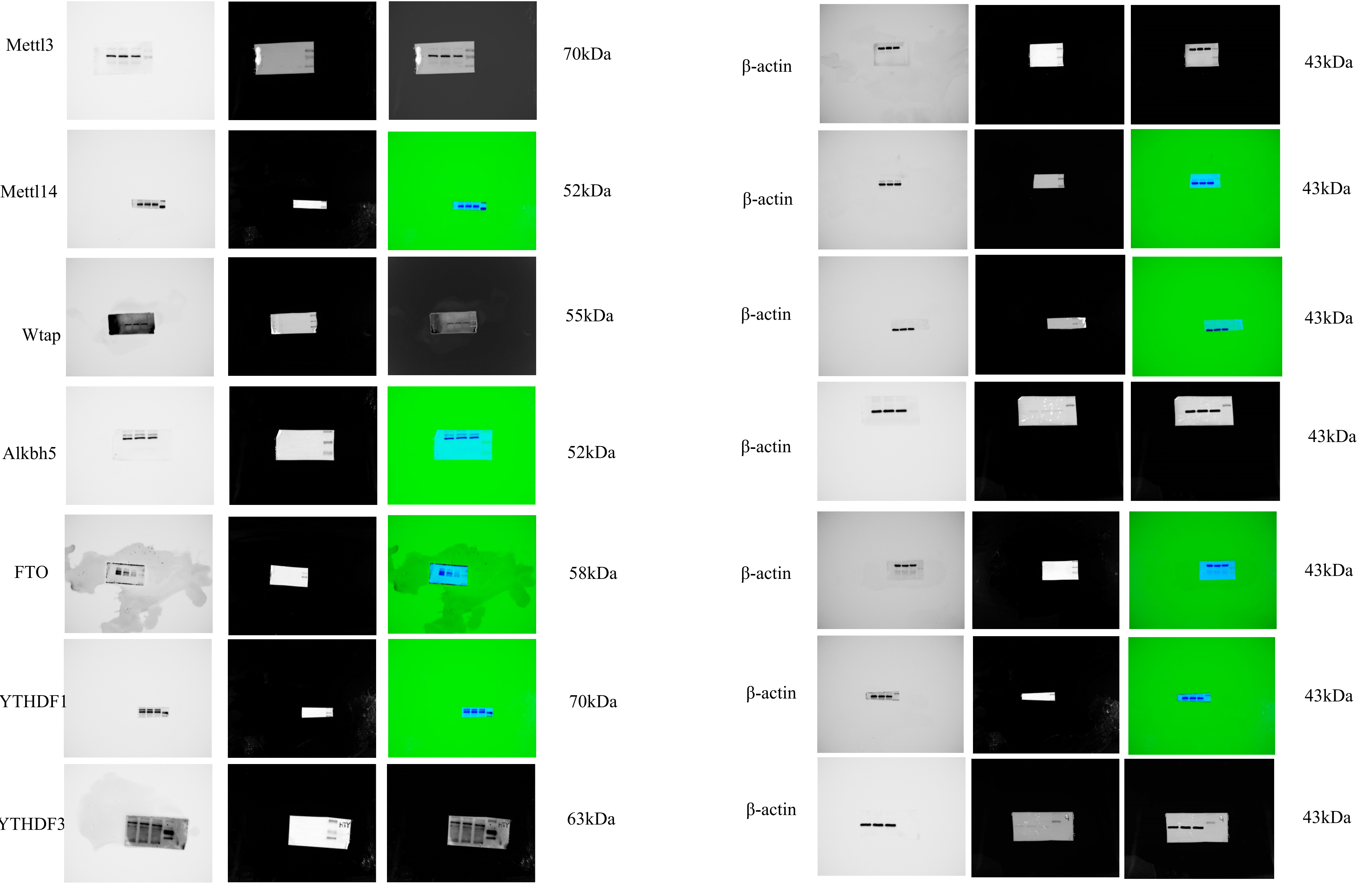
Mettl3
70kDa
Mettl14
52kDa
Wtap
55kDa
Alkbh5
52kDa
FTO
58kDa
YTHDF1
70kDa
YTHDF3
63kDa
β-actin
43kDa
β-actin
43kDa
β-actin
43kDa
β-actin
43kDa
β-actin
43kDa
β-actin
43kDa
β-actin
43kDa
