# Supplementary figures and images for: N6-methyladenosine demethylase FTO regulates synaptic and cognitive impairment by destabilizing PTEN mRNA in hypoxic-ischemic neonatal rats
Source: Cell Death Dis. 2023 Dec 13;14(12):820. doi: 10.1038/s41419-023-06343-5 (PMC10719319; doi:10.1038/s41419-023-06343-5)

A

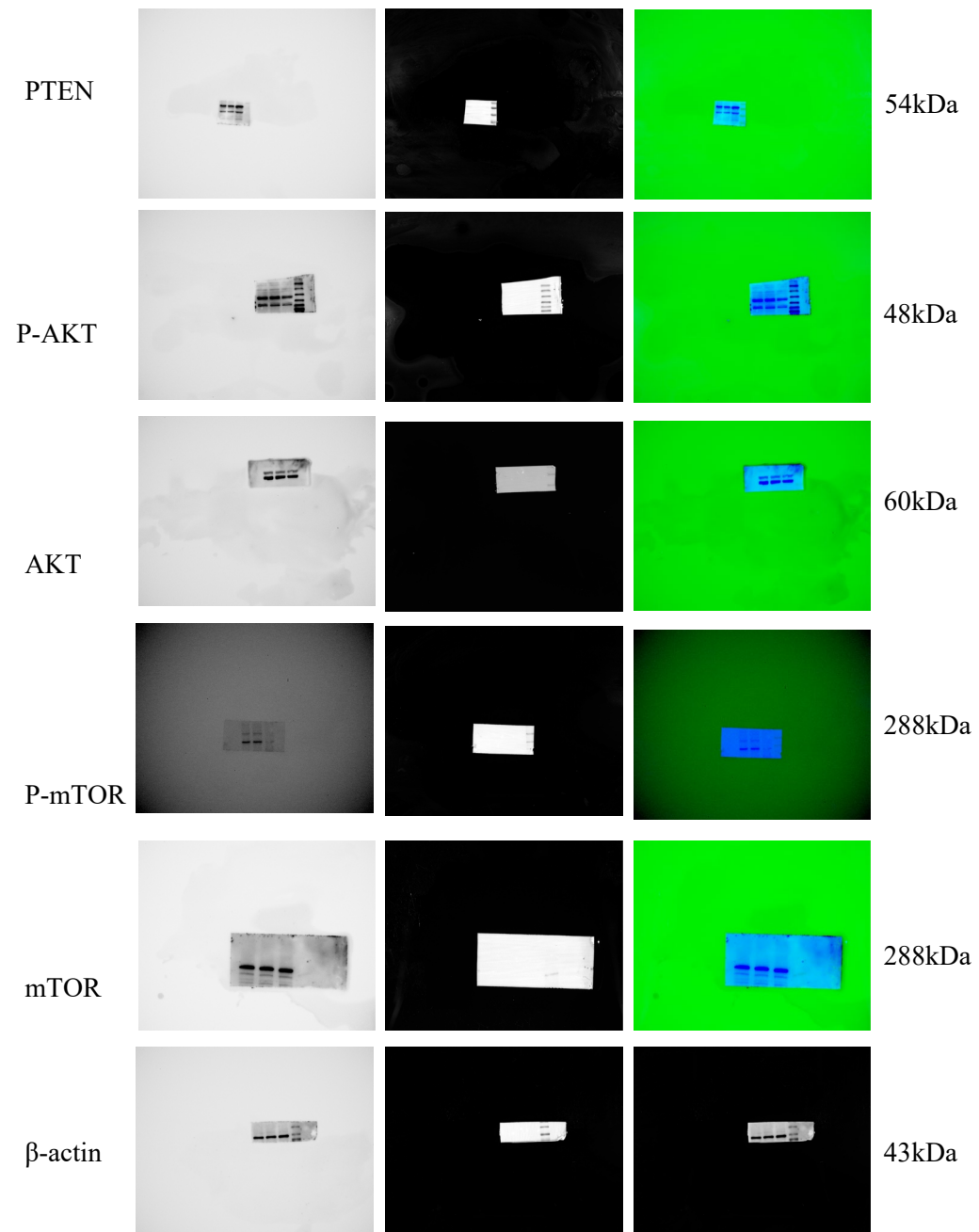

B

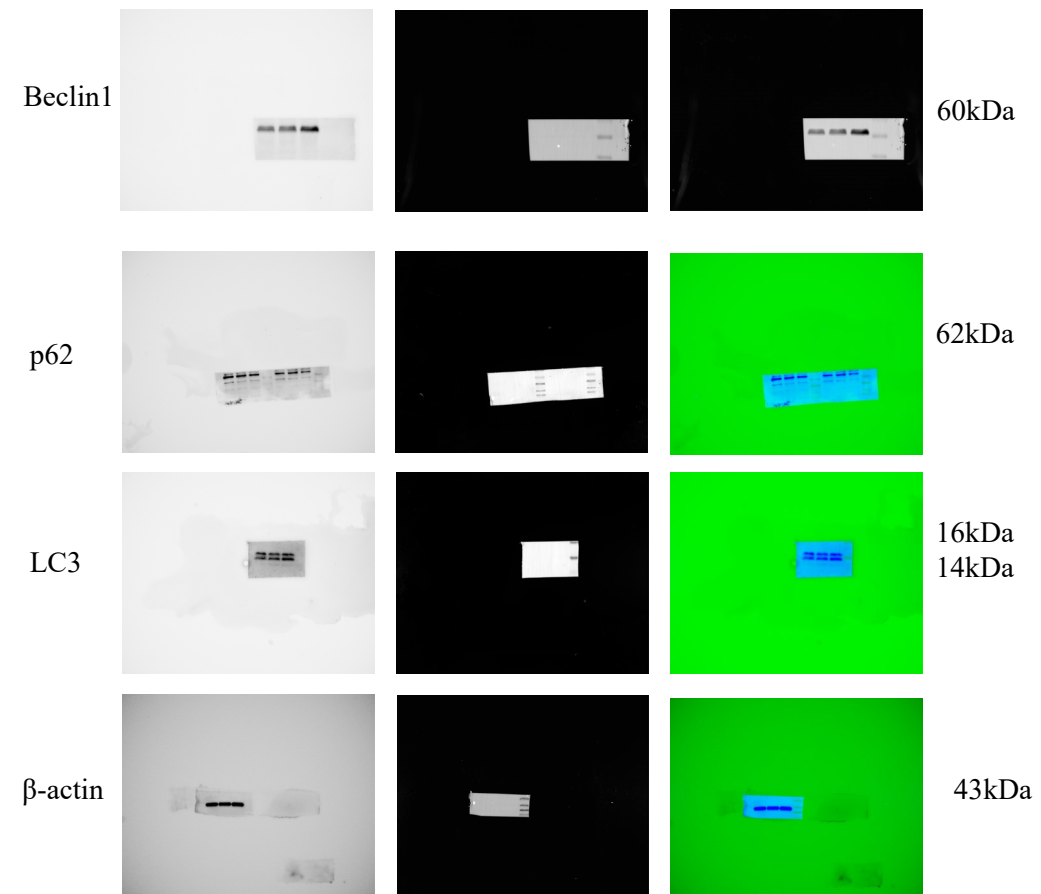

Supplement: Supplementary file 4 — F4 western blot original data [file 41419_2023_6343_MOESM4_ESM.pdf]

C

FTO

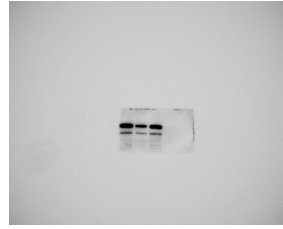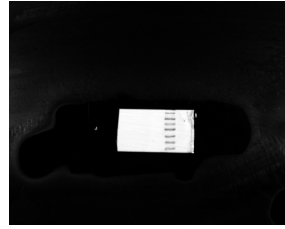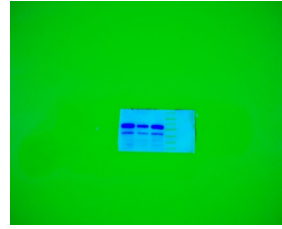

58kDa

$\beta$ -actin

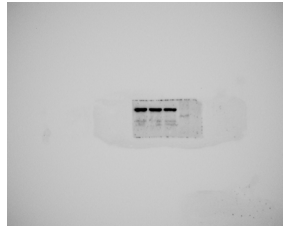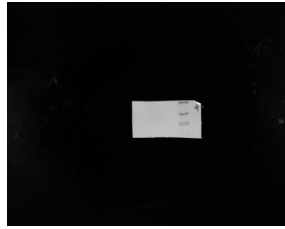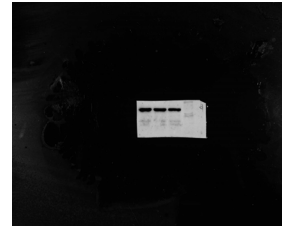

43kDa

G

FTO

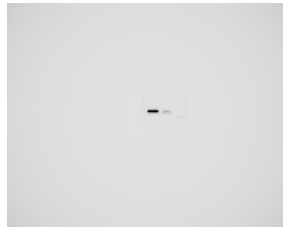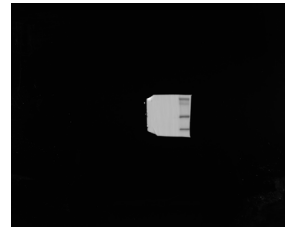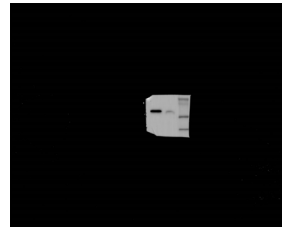

58kDa

$\beta$ -actin

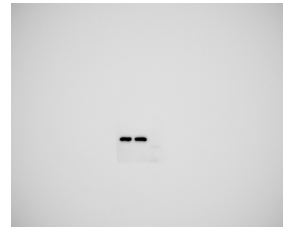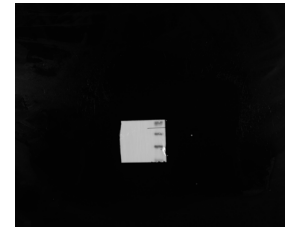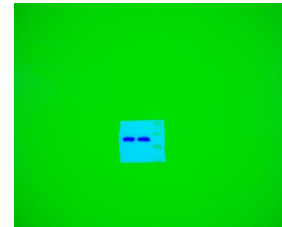

43kDa

Supplement: Supplementary file 5 — F5 western blot original data [file 41419_2023_6343_MOESM5_ESM.pdf]

D

PTEN

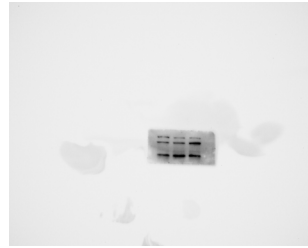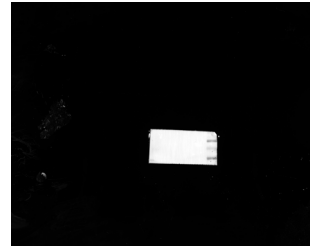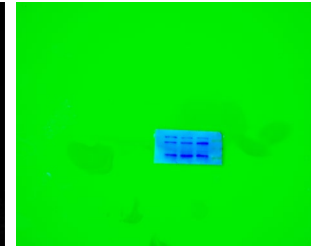

54kDa

$\beta$ -actin

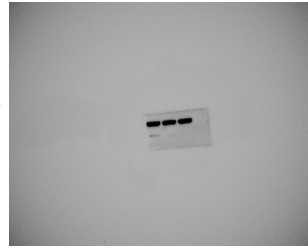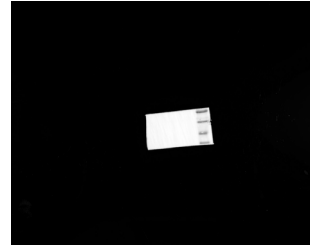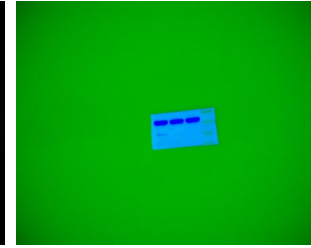

43kDa

Supplement: Supplementary file 6 — F6 western blot original data [file 41419_2023_6343_MOESM6_ESM.pdf]

A

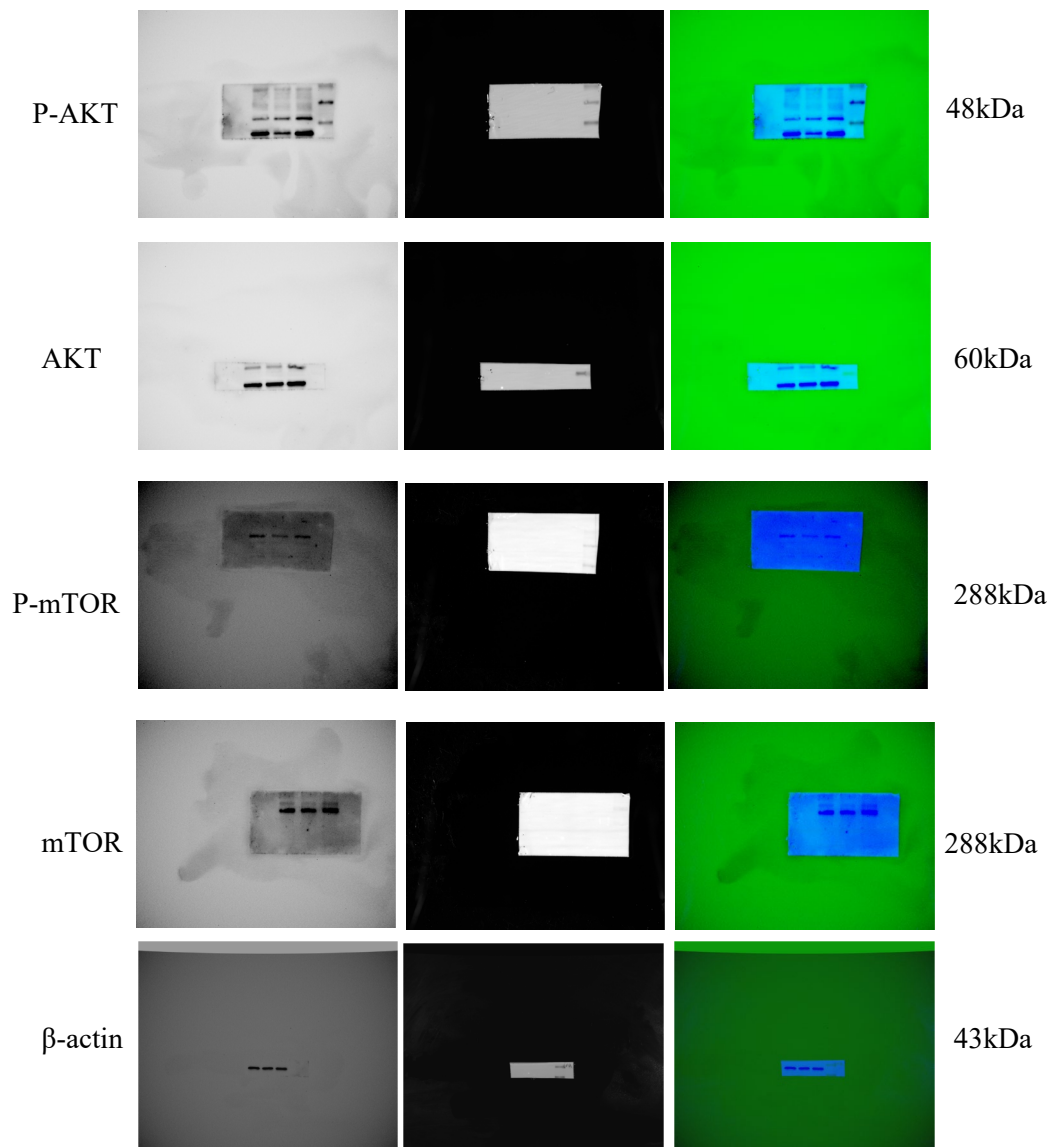

D

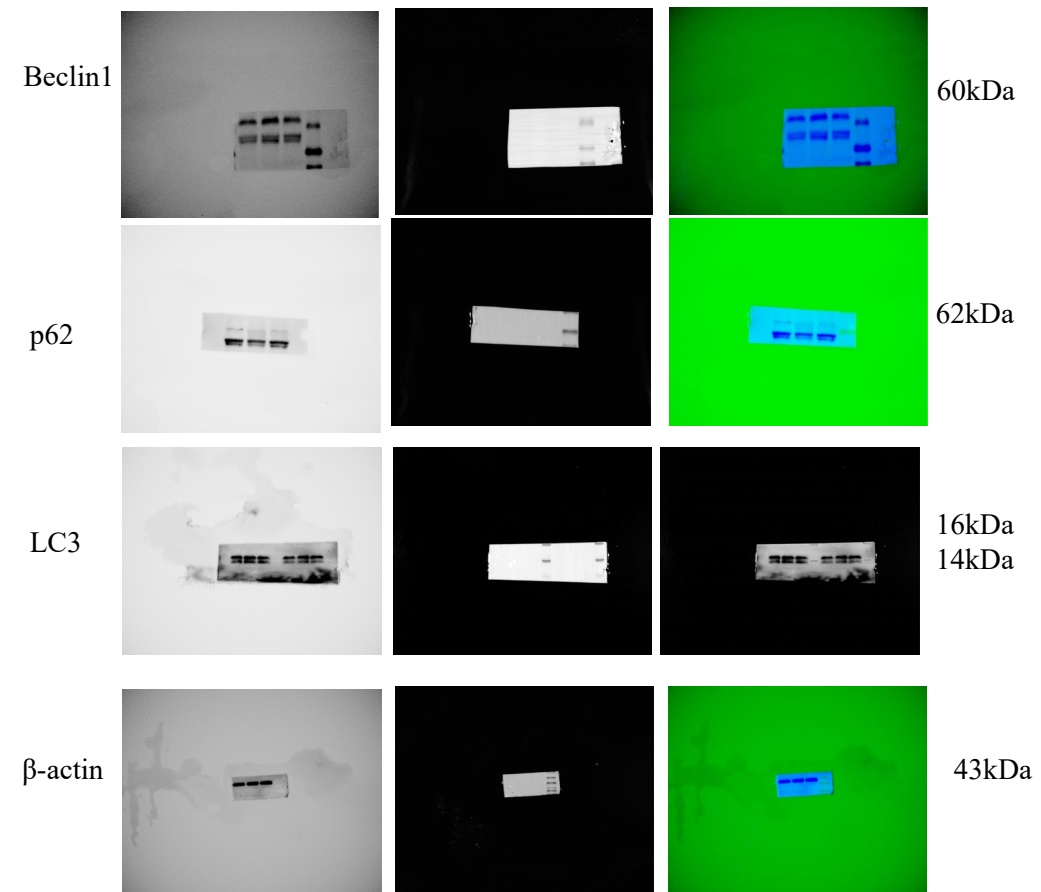

Supplement: Supplementary file 7 — F7 western blot original data [file 41419_2023_6343_MOESM7_ESM.pdf]

C

PSD95

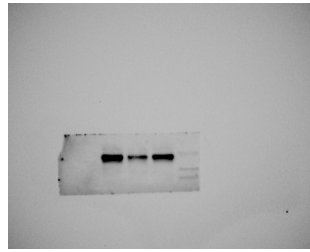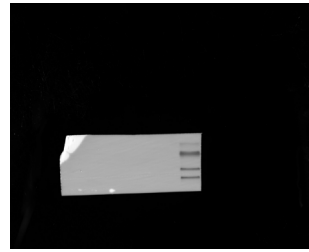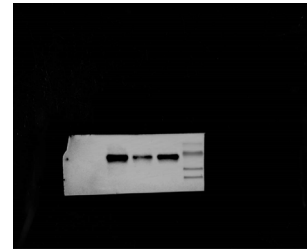

95kDa

$\beta$ -actin

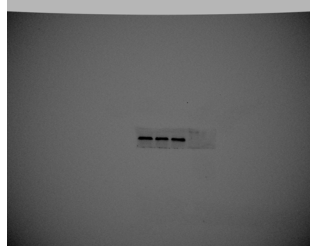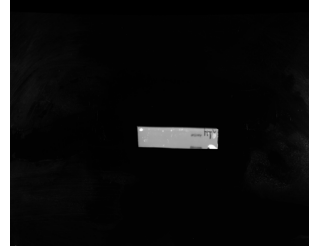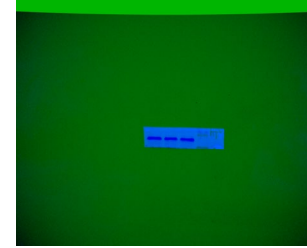

43kDa

synaptophysin

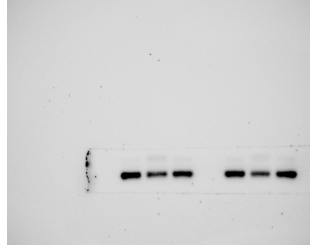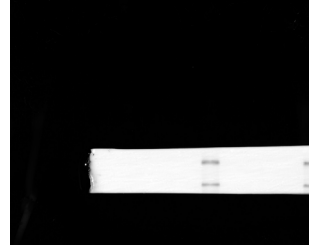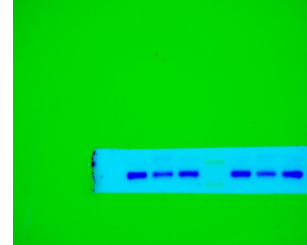

38kDa

Supplement: Supplementary file 8 — F8 western blot original data [file 41419_2023_6343_MOESM8_ESM.pdf]

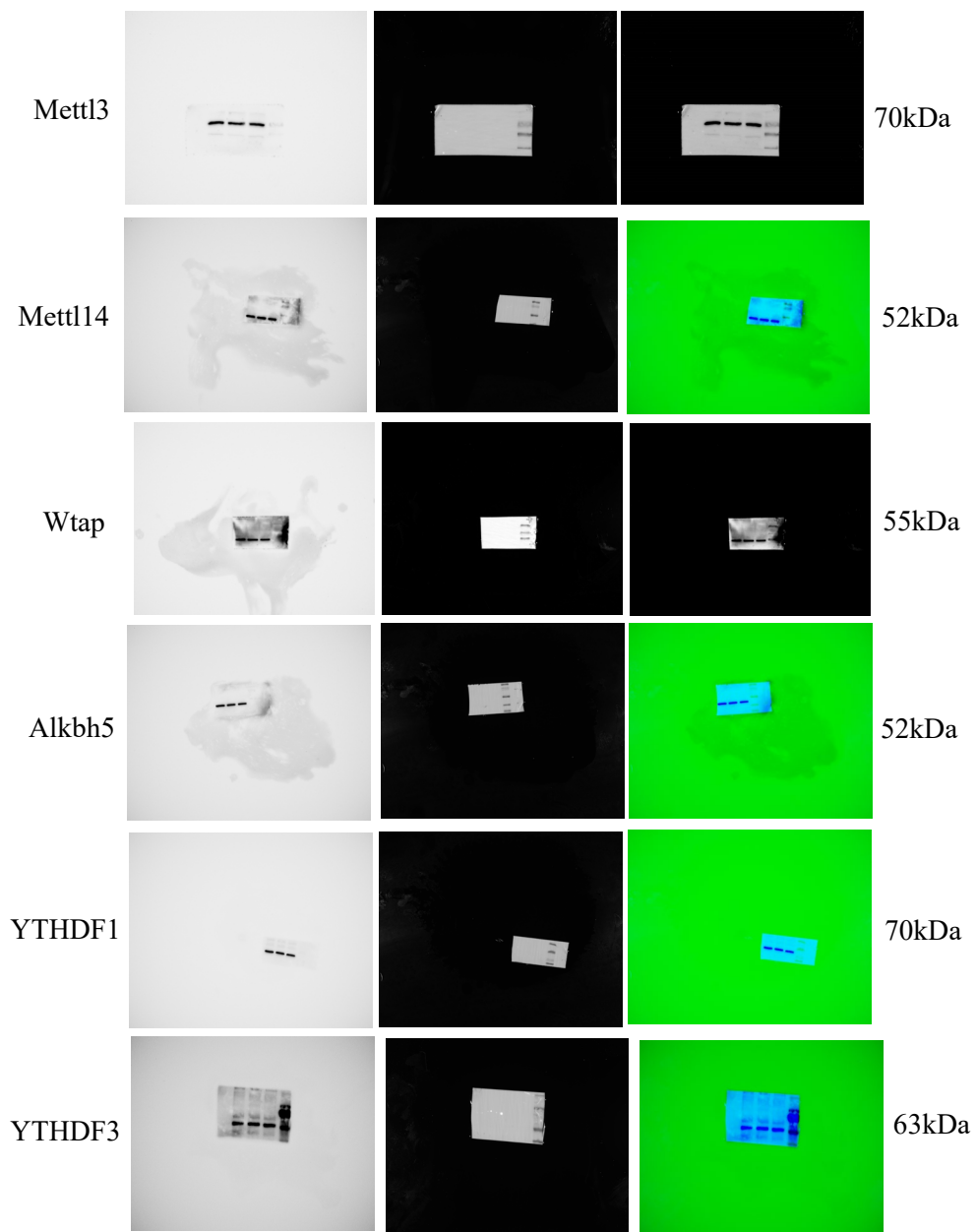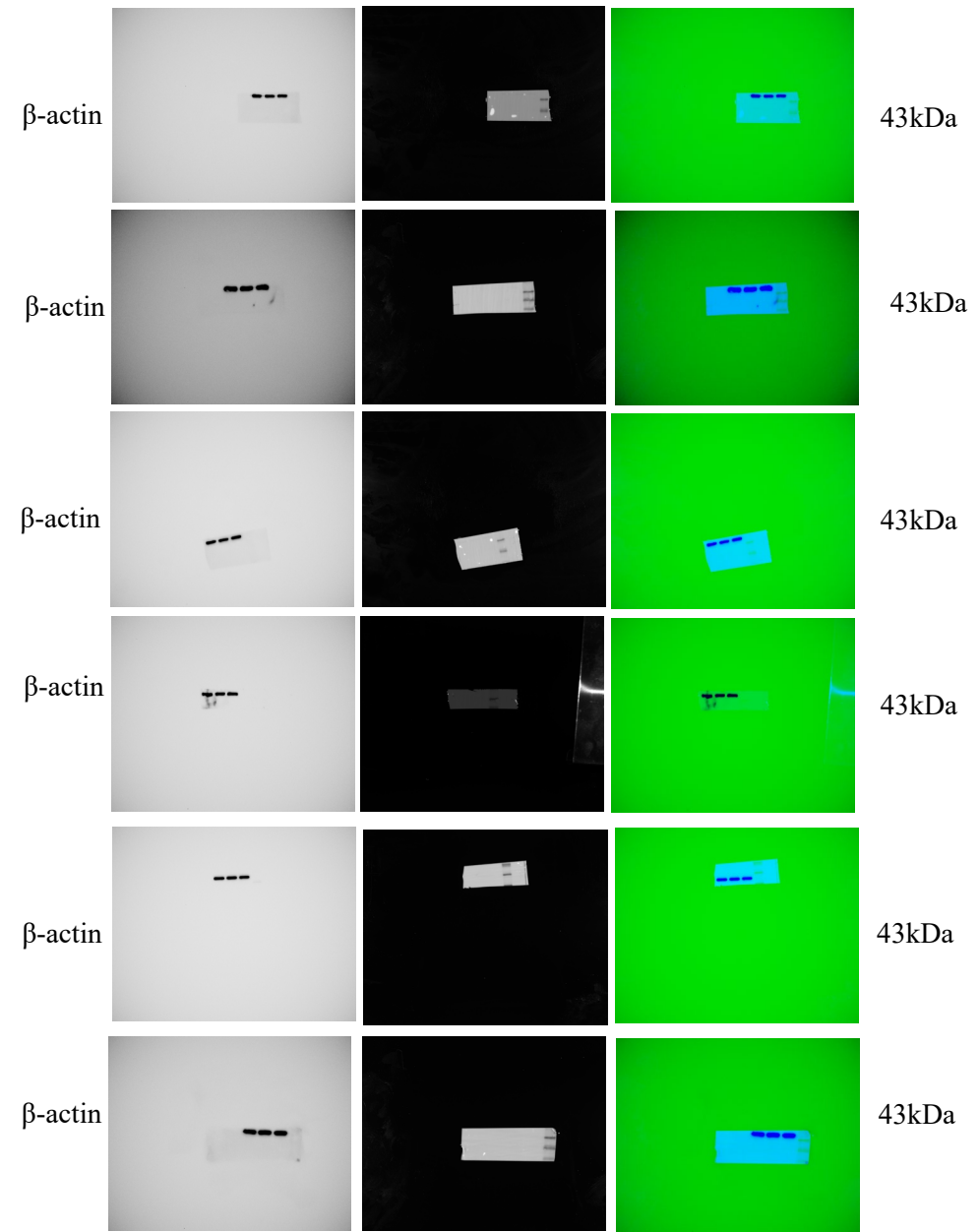

Supplement: Supplementary file 9 — FS1 western blot original data [file 41419_2023_6343_MOESM9_ESM.pdf]
